# *Toxoplasma gondii* Seroprevalence and Trends in Women Presenting for *Toxoplasma* Screening in South-West Romania

**DOI:** 10.3390/microorganisms11082057

**Published:** 2023-08-10

**Authors:** Cristiana Luiza Radoi, Ovidiu Mircea Zlatian, Maria Balasoiu, Lucian Giubelan, Andreea Cristina Stoian, Livia Dragonu, Alexandru Neacsu, Dominic Gabriel Iliescu

**Affiliations:** 1 Doctoral School, University of Medicine and Pharmacy of Craiova, 200349 Craiova, Romania; luizacristianaradoi@gmail.com (C.L.R.); alex.neacsu1210@gmail.com (A.N.); 2 Medical Laboratory, County Clinical Emergency Hospital of Craiova, 200349 Craiova, Romania; balasoiu.maria@yahoo.com; 3 Microbiology Department, University of Medicine and Pharmacy of Craiova, 200349 Craiova, Romania; 4 Infectious Diseases Department, University of Medicine and Pharmacy of Craiova, 200349 Craiova, Romania; ligiubelan@yahoo.com (L.G.); andreea_plr@yahoo.com (A.C.S.); livia_dragonu@yahoo.com (L.D.); 5 “Victor Babes” Infectious Diseases and Pneumophtisiology Clinical Hospital, 200349 Craiova, Romania; 6 Obstetric and Gynecology Department, County Clinical Emergency Hospital of Craiova, 200349 Craiova, Romania; dominic.iliescu@yahoo.com; 7 Obstetric and Gynecology Department, University of Medicine and Pharmacy of Craiova, 200349 Craiova, Romania

**Keywords:** Toxoplasma gondii, seroprevalence, childbearing age women

## Abstract

Toxoplasmosis, caused by the obligate intracellular protozoan parasite Toxoplasma gondii (*T. gondii*), is a globally prevalent zoonotic disease with potentially severe implications for immunocompromised individuals, pregnant women, and their fetuses/children. This study examined the prevalence of anti-*T. gondii* IgM and IgG antibodies in two groups of childbearing age women, including 653 participants in Group 1 (2013–2016) and 3221 participants in Group 2 (2019–2022). Our results revealed a decrease in the overall positivity rate of anti-*T. gondii* IgM antibodies from 2.32% to 1.06%, suggesting improved public health interventions over time. However, there were variations among different age groups and between rural and urban environments, with a significant decrease in urban areas across all age groups from Group 1 to Group 2. Regarding anti-*T. gondii* IgG antibodies, we did not observe a significant change in the seropositivity rate between the two groups. In the rural population with an age group over 35 years, we observed the highest positivity rate in Group 2. This study provided information on the risk factors and burden of toxoplasmosis in women of childbearing age with data that can be valuable to public health policies and the planning of healthcare measures for effective toxoplasmosis management.

## 1. Introduction

Toxoplasmosis is a parasitic infection caused by *Toxoplasma gondii* (*T. gondii*), an obligate intracellular protozoan parasite that infects a wide range of warm-blooded animal species, including cats, who can transmit the infection to humans [1,2]. This zoonotic disease has gained considerable attention due to its global prevalence and potential to cause severe complications in certain populations [3].

In immunocompetent individuals, *T. gondii* infection can be asymptomatic or exhibit mild flu-like symptoms [4]. In immunocompromised patients, for example, infected with HIV/AIDS or treated with immunosuppressive drugs, life-threatening conditions can occur, such as encephalitis and severe pneumonia [5,6,7].

Pregnant women are particularly at risk, as the fetus can be infected with *T. gondii,* leading to congenital toxoplasmosis, which can be manifested as a miscarriage, stillbirth, or severe neurological and ocular abnormalities in newborns [8,9]. The incidence of congenital toxoplasmosis is estimated to be 0.1 per 1000 live births [10]; the risk is higher if the mother has suggestive symptoms, such as lymphadenopathy [11]

The severity of fetal infection is inversely correlated with gestational age, with significant implications for fetal development if transmission occurs during the first trimester [8,9,12]. Beyond neurological disorders, toxoplasmosis can result in chorioretinitis (blurred vision and eye pain) [3,9]. Recent studies have reported a link between latent toxoplasmosis and behavioral or psychiatric alterations [13].

Several factors such as sanitation levels, temperature, humidity, host susceptibility as well as exposure to soil and domestic animals such as cats contribute toward the prevalence of toxoplasmosis [14,15]. Globally, seroprevalence in humans varies widely, from 10% to 90%, depending on geographical and cultural factors. [13,14,16,17,18,19,20,21,22,23,24,25].

For instance, highly endemic regions in certain parts of Africa have reported up to 90% seroprevalence, while some European populations have reported rates up to 60% [9]. A study of the seroprevalence of antibodies against TORCH agents (*Toxoplasma gondii*, Rubella virus, Cytomegalovirus, Herpes simplex virus, and other agents) shows that about 22.5% of the US population has evidence of previous exposure to *T. gondii* [26] indicating the need for continuous surveillance at both global and local levels for effective management strategies.

The economic burden of toxoplasmosis is substantial, with direct and indirect costs relating to diagnosis, treatment, and potential long-term sequelae [13]. These costs underscore the importance of increased awareness, accurate diagnostic methods, and effective prevention strategies to mitigate the impact of this parasitic infection [14].

### Aim

Continuous surveillance at both global and local levels is essential for effective management. Accurate prevalence data of toxoplasmosis in the Romanian population is crucial to effectively prioritize public health policy, allocate funding for interventions and plan healthcare measures with the aim of reducing the burden of toxoplasmosis on maternal and neonatal health. To the best of our understanding, Romania lacks robust epidemiological studies supported by rigorous statistical controls. The collection of epidemiological data has predominantly been conducted retrospectively, meaning they are deprived of requisite calculations for statistical significance.

Our study sought to estimate *T. gondii* seroprevalence in fertile women from the South-West region of Romania using a rigorous academic approach. Furthermore, we aimed to investigate the relationship between demographic factors (such as area of residence and age) and *T. gondii* seroprevalence in two time periods (2013–2016, 2019–2022).

## 2. Materials and Methods

Study design. The study was retrospective, observational, and cross-sectional and was carried out on two groups of childbearing-age women who presented consecutively at the County Clinical Emergency Hospital of Craiova, Romania, in two time periods: between 2013 and 2016 (Group 1) and 2019 and 2022 (Group 2). Group 1 comprised 653 participants, while Group 2 had 3221 participants.

Participants. The study participants were Romanian women of childbearing age with residence in counties within the South-West region of Romania (Dolj, Gorj, Olt, Mehedinti, Valcea). The women were addressed to the hospital for the routine screening of antibodies against TORCH agents.

Data collection. For each participant, we recorded the age, area of residence, and the results of laboratory testing for anti-*T. gondii* IgM and IgG antibodies.

Laboratory testing for anti-*T. gondii* IgM and IgG antibodies. *T. gondii* serology was assessed during the first-trimester standard evaluation. Negative IgG cases were reassessed during the second trimester (24–25 gestational weeks) and third trimester (30–33 gestational weeks). The titers of IgM and IgG antibodies were determined by two different methods. For Group 1 (2013–2016), we used an immune electro-chemical luminescence assay with the analyzer Cobas E601 (Roche Diagnostics, Basel, BS, Switzerland), and for Group 2, we used an immune chemiluminescence assay with the analyzer Architect i1000 (Abbott, Abbott Park, IL, USA). We used commercial reagents provided by the manufacturers of these analyzers (Roche and Abbott). The analyzers’ proprietary software (version 0601) classified the tests as positive or negative according to cut-off values.

Therapeutic protocol. Pregnant women or women that planned pregnancy with confirmed *T. gondii* infection were referred to the Department of Infectious Diseases, where oral spiramycin at 1 g and 3 times daily were recommended to prevent transmission to the fetus. The regimen was started as soon as possible after serological diagnosis and continued throughout pregnancy. In pregnant women, amniocentesis was performed at 20 to 24 gestational weeks to investigate fetal infection. Ultrasound monitoring for the presence of fetal infection markers was indicated.

Statistical analysis. Data were exported from the hospital software HIPOCRATE (version H3 Concept) in the statistical software STATA 17 (Statacorp Ltd., College Station, TX, USA). Numerical data were expressed as the mean (standard deviation), and count data were expressed as the count (percentage). We used the Chi-squared test to assess the differences between Group 1 and Group 2, using a statistical significance level of *p* < 0.05. The age differences between Group 1 and Group 2 were tested using Student’s t-test as age was normally distributed in both groups.

We used the software Adobe Illustrator version 27.7 (Adobe Inc, San Jose, CA, USA) to construct a map of toxoplasmosis prevalence in Dolj County, Romania, based on publicly available county maps, in which we only included towns with more than 20 subjects tested. The areas where we had prevalence data were colored according to the prevalence level.

Ethical issues. This study was approved by the Committee of Ethics and Academic and Scientific Deontology, Craiova, Romania (approval no. 84/16.09.2020). The study adheres to both ethical guidelines and the legal requirements of the country where it was conducted.

## 3. Results

### 3.1. Anti-Toxoplasma gondii IgM Antibodies

Demographic characteristics. Group 1 (2013–2016) consisted of 602 females, with fewer females from rural areas (34.21%). Group 2 (2019–2022) included 3014 females, with a higher proportion from rural areas (56.54%). The mean age remained consistent between the two groups (27.79 vs. 27.62, *p* = 0.537) (Table 1). Both groups had the highest number of tested women in the 26–30 years age group. Group 2 showed a slightly higher number of tested women in the >35 years age group (Table 2).

*Seroprevalence results*. The highest seroprevalence was noted in women below 20 years in Group 1 (3/57, 5.26%) and in those over 35 years in Group 2 (6/140, 1.76%). In Group 1, the 26–30 years age group presented with the lowest seroprevalence in rural areas (1.49%), while in Group 2, the lowest seroprevalence was in the 21–25 years age group in urban areas (0.44%) (Table 2 and Table 3).

We observed a significant decrease in the overall positive results for anti-*T. gondii* IgM antibodies from 2.32% in Group 1 to 1.06% in Group 2 (*p* = 0.012) (Table 2). In both Group 1 and Group 2, seroprevalence decreased from rural areas to urban areas (2.90% vs. 2.02% and 1.40% vs. 0.61%, respectively).

A significant decline in prevalence was observed in urban areas across all age groups from Group 1 to Group 2 (from 2.02% to 0.61%, *p* = 0.011), with a similar trend in rural areas (from 2.91% to 1.40%, *p* = 0.099), though this was not statistically significant (Table 2). In Group 1, the prevalence was higher in urban areas for women under 30 years, while in Group 2, rural areas showed a higher prevalence.

A descending trend in positive results was observed in women under 20 years between the two groups, both in rural (2.70% vs. 1.80%) and urban areas (10.00% vs. 0.97%, *p* = 0.017) (Table 2 and Table 3) and in women aged 31–35 years from rural areas (from 6.89% in the 2013–2016 group to 0.69% in the 2019–2022 group).

### 3.2. Anti-Toxoplasma gondii IgG Antibodies

*Demographic characteristics.* Group 1 included 603 participants (34.00% rural), while Group 2 included 2901 participants (56.12% rural). The proportion of participants that were from rural areas increased with statistical significance from the 2013–2016 group to the 2019–2022 group (*p* < 0.001) (Table 4). Age distribution differed slightly between the groups.

*Seroprevalence results.* Both Group 1 and Group 2 had the highest seroprevalence in rural areas in women aged over 35 years (75.00% and 62.12%, respectively). The lowest seroprevalence in Group 1 was in women aged 31–35 from rural areas (13.79%), while in Group 2, it was in women from urban areas aged 26–30 (29.80%) (Table 5).

Overall, a slight increase in seroprevalence from 37.81% in 2013–2016 to 38.54% in 2019–2022 was observed. The seroprevalence showed a slight increase in urban areas compared with rural areas in Group 1 (from 36.59% to 38.44%), while in Group 2, the seroprevalence decreased from rural to urban areas (from 42.44% to 33.54%). In rural areas, there was a slight increase in IgG seroprevalence between the two time periods from 36.59% to 42.44% (*p* = 0.109), while in urban areas, we observed a slight decrease from 38.44% to 33.54% (*p* = 0.073).

In young women (< 20 years age group), we noticed a statistically significant decrease in IgG seroprevalence from 50.00% to 31.73% (*p* = 0.006) between the two time periods and in the 21–25 years age group in rural areas (*p* = 0.012). Interestingly, in women aged 31–35 years from rural areas, we observed an important increase in seroprevalence from 13.79% to 47.96% (*p* < 0.001) (Table 5 and Table 6).

### 3.3. Seroprevalence of Anti-T. gondii IgM Antibodies in Immunized Childbearing Age Women

In Group 1, the odds of having an anti-*T. gondii* IgM positive status was significantly higher in the anti-*T. gondii* IgG negative group compared to the anti-*T. gondii* IgG positive one (odds ratio = 10.81, 95% CI = 2.36–100.00, *p* < 0.001). In Group 2, the odds of anti-*T. gondii* IgM positivity was found to be significantly higher among women who tested negative for anti-*T. gondii* IgG (odds ratio = 40.77, 95% confidence interval = 6.65–1674.49, p < 0.001) (Table 7).

### 3.4. Maping of Toxoplasmosis Prevalence in Dolj County between 2019–2022

**Figure 1:**
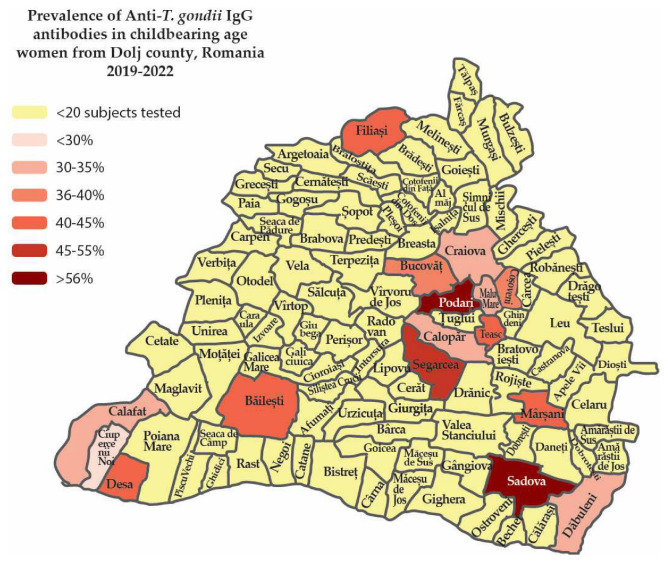
IgG anti-*T. gondii* seroprevalence in childbearing women—spread in Dolj county, Romania 2019–2022.

As our hospital from Craiova City tested childbearing-age women from various settlements in Dolj County, we analyzed the results based on the town of residence (Table 8, Figure 1).

The biggest seroprevalence was recorded in Sadova (57.57%) and Podari (58.97%) settlements, followed by Segarcea (45.83%), with a lower prevalence in Teasc (42.85%), Filiași (41.93%), Mârșani (40.90%), Desa. (40.90%) and Băilești (40.00%). The lowest prevalence was reported in Ciupercenii Noi (18.18%) (Table 8, Figure 1).

## 4. Discussion

The insights from our study contribute valuable information to the understanding of *T. gondii* epidemiology, highlighting the need for targeted interventions and continuous surveillance. Our findings reflect a dynamic pattern in the seroprevalence of *T. gondii* infection with notable shifts in its prevalence across different demographics. In general, antibody levels can vary significantly depending on the region and age group of the subjects [8,14]. Other studies reported significant differences between urban and rural populations in terms of their exposure to certain pathogens [27,28].

In our study, the number of women that tested for *T. gondii* infection markedly increased from 2013–2016 to 2019–2022 due to the increase in the medical information of patients and the progress in laboratory technology associated with a reduction in testing costs that have become affordable for the average pregnant women [29,30].

### 4.1. Anti-Toxoplasma gondii IgM Antibodies

In our study, the anti-*T. gondii* IgM antibodies’ seroprevalence was 2.32% in the 2013–2016 group and 1.06% in the 2019–2022 group, showing a marked decrease (Table 2). This could be due to an improvement in the socioeconomic status of the Romanian population [31].

Across Europe, there was also a reported decrease in seroprevalence in a study performed in Italy, from 1.60% in 2010 compared to 0.98% in 2007 [32]. A study performed in France reported a seroprevalence rate that declined from 0.75% in 1980 to 0.35% in 2000 and further to 0.24% in 2010 [33].

In Romania, a study from its Western region reported a slight increase in anti-*T. gondii* IgM seroprevalence from 2008–2010 (0.80%) to 2015–2018 (1.10%) [34]. A study performed in a big city in Northern Romania showed an IgM seroprevalence of 7.21% [35]. A possible explanation for this high prevalence is the higher testing rate for anti-*T. gondii* antibodies in the big cities, where access to testing is facile.

Our study found in the 2013–2016 group, the highest prevalence (5.26%) of anti-T. gondii IgM antibodies in the age group under 20 years, while in the 2019–2022 group, the highest prevalence (1.76%) was in the >35 years age group. A study from the Moldavia area of Romania detected an IgM seroprevalence of 7.35% in the 25–34 age group and 9.09% in the >35 years age group [35]. We can suppose that women over 35 years of age are more informed to perform all the necessary pregnancy screening tests, especially in recent years, resulting in a higher number of TORCH tests, as reported by other authors [36].

In both groups, the seroprevalence was higher in rural areas compared with urban areas, while a study from Brazil [27] reported no association between anti-*T. gondii* IgM seropositivity and rural residence or age.

Possible explanations for the declined seroprevalence in urban areas include decreased exposure to cats, higher socioeconomic status, modern and centralized sanitation systems, and the rigorous monitoring of water quality [10].

The seropositivity for IgM antibodies cannot be interpreted as an acute *T. gondii* infection because is well documented that IgM antibodies persist in individuals for a duration of 40 to 50 weeks after acute infection [37], while studies involving fertile women have reported a range of 25 to 65 weeks [17]. For congenitally infected neonates, the decay in IgM antibodies occurred more rapidly compared to adult individuals, although some remained positive for as long as 30 weeks postpartum [38].

### 4.2. Anti-Toxoplasma gondii IgG Antibodies

Our study showed a prevalence of anti-*T. gondii* IgG antibodies at 37.81% in the 2013–2016 group and 38.54% in the 2019–2022 group.

Various studies across Europe have examined the variability of anti-*T. gondii* IgG prevalence among pregnant women populations [13,16,17,18,19,20,21,22] from 8.2% in Switzerland [24] to 59% in Germany [23]. A study on pregnant women performed in Iraq found a total IgG seroprevalence of 33.7%, and the maximum prevalence (64%) was recorded in the 21–30 years of age group [28].

Information regarding the prevalence of *T. gondii* in the human population in Romania is scarce [8]. Most serological surveys rely on convenience samples [9]. One exception is a study performed by Coroiu et al. in 2009 [39], which used a stratified sampling strategy from the general population located in 11 counties from central and North-West Romania (4.6 million people). From the 1155 samples tested, anti-*T. gondii* IgG antibodies were present in 59.48%, with a higher prevalence in rural areas (63.68%) vs. urban areas (55.12%), without significant gender differences but with an estimated annual risk in young people of 4.5% [39].

Research from Western Romania indicated a decrease in *T. gondii* infections by approximately five percentage points (from 43.79% to 38.81%) among pregnant women over ten years between 2008–2010 and 2015–2018 [40].

Another investigation in Western Romania reported a prevalence of 64.8% in 2015 [41]. Subsequent research during 2020 and 2022 revealed prevalence rates ranging between 36.48% and 55.8% in Western Romania [8,34,39,40,41,42,43,44]. The lowest prevalence was observed in a study from the Bihor area (Western Romania) [45], which reported a rate of 25.4% among pregnant women who sought consultation at infectious disease clinics. Interestingly, in childbearing-age women from Western Romania, another study from the same area [42] also indicated a relatively low prevalence of 36.48% compared with another study from the Banat (Western Romania) area, which found a prevalence of 41.16% [46].

A study on pregnant women from Northern Romania [35] showed a prevalence of IgGs of 32.83% in the 25–34 age group and 37.83% in the >35 years age group, while in our study, the highest prevalence was recorded in the <20 years age group (50.00%) and in the over 35 years of age group it was 35.19%. Another study from Western Romania supported our research showing that the highest prevalence of anti-*T. gondii* IgG antibodies were in women aged between 20 and 30 years [39].

Regarding the area of residence, in Group 1, our results showed similar seroprevalences in rural and urban areas, while in Group 2, the seroprevalence was higher in rural areas. Supporting our results, a study from Poland [47] found that women living in rural areas had three times higher odds of being infected with *T. gondii* compared with urban areas. Another study from Brazil also found an OR of 3.46 for anti-*T. gondii* IgG in rural vs. urban areas [27].

The differences in seroprevalence between studies from various regions could be explained by the heterogeneity of the studied groups. There are differences in population structure by age and area of residence, as well as other factors like education level that influence the prevalence of toxoplasmosis. Additionally, the patient populations addressing private centers and public hospitals (where testing is free) are different in terms of demographic and socioeconomic factors [48].

### 4.3. Seroprevalence of Anti-T. gondii IgM Antibodies in Immunized Childbearing Age Women

Our findings suggest that anti-*T. gondii* IgG status is a significant predictor of anti-*T. gondii* IgM status among women, and screening for anti-*T. gondii* IgG might be useful in identifying women at risk for *T. gondii* infection during pregnancy. Therefore, IgG-negative women should be screened and re-screened for *T. gondii* infection. We cannot, however, generalize that the positive test for IgG contraindicates the IgM test because there is a non-negligible possibility for IgG seropositive women to also have anti-*T. gondii* IgM antibodies [37]. A definitive diagnosis can only be conducted through the IgG avidity test or PCR test for CMV DNA [48].

These results bear significant implications for devising public health policies and interventions, especially within communities where the prevalence of *T. gondii* is relatively low. Additionally, data from our study enhanced the existing epidemiological comprehension of *T. gondii* in pregnant women, paving the way for more informed and targeted preventative and treatment measures against this infection.

### 4.4. Maping of Toxoplasmosis Prevalence in Dolj County between 2019–2022

There was a notably high seroprevalence of anti-*T. gondii* IgG antibodies in fertile women tested from various settlements in Dolj county, South-West Romania. Interestingly, the toxoplasmosis prevalence was higher in the settlements near Craiova city than in the city of Craiova, which could be explained by easy access to Craiova hospital due to short distance bus lines.

The higher seroprevalence in the rural areas around Craiova might be due to factors specific to rural areas [28,47], such as the higher number of cats per household, lower adherence to hygiene measures, and a lower level of education [49]. As women from rural areas are at a higher risk for *T. gondii* infection, targeted intervention strategies should be considered in these areas, including health education programs about toxoplasmosis risk factors and prevention methods.

Studies have demonstrated a positive correlation between toxoplasmosis prevalence and factors such as drinking unfiltered water and contact with animals and soil [50,51,52,53]. This implies that people, especially farm workers in rural areas, are more exposed to sources of toxoplasmosis infection [27,51,53].

#### 4.4.1. Limitations

The present study has several limitations. Our sample was a convenience sample; specifically, we took all women addressing for the serological testing of anti-*T. gondii* antibodies in a single center. Although most of the women performed both IgM and IgG tests, some had tested either only the IgG or IgM antibodies, which explained the difference between the number of women tested for IgM and IgG. We did not analyze all the factors that could increase the risk of acquiring toxoplasmosis because we did not administer questionnaires to these women. We only analyzed the demographic factors that could influence the risk of acquiring toxoplasmosis. For example, several studies showed that eating raw or undercooked meat was a significant risk factor [28,47], and there probably exist other risk factors that can confound the group differences we found.

In our study, we observed a shift toward more participants from rural areas in the second time period, which could have implications for the generalization of this study’s findings. It is also worth noting that other factors, such as changes in recruitment methods or eligibility criteria, could have influenced these results; therefore, it is important to consider the context and limitations of the study when interpreting these findings [36].

#### 4.4.2. Future Directions

Addressing the high prevalence of toxoplasmosis in childbearing-age women in Dolj County requires a multifaceted approach that includes increased screening, targeted health education, the promotion of behavioral changes, and preconception counseling.

The fact that the seroprevalence of anti-*T. gondii* IgG antibodies were rather low could also be regarded as there is a high proportion of women at risk for acute toxoplasmic infection.

Further research is needed to better understand the factors influencing the prevalence of toxoplasmosis and to develop effective strategies for prevention and control in different regions and populations, particularly in populations at a higher risk of infection, such as pregnant women.

## 5. Conclusions

The seroprevalence of anti-*T. gondii* IgM antibodies in tested childbearing-age women, including either positive or negative for anti-*T. gondii* IgG antibodies showed a marked decrease from 2013–2016 to 2019–2022, which is more significant in urban areas and in 21–25 years and 31–35 years age groups. We found that anti-*T. gondii* IgG immunization is a significant protection factor against toxoplasmosis infection and was detected by anti-*T. gondii* IgM in women; therefore, screening for anti-*T. gondii* IgG might be useful in identifying women at risk for *T. gondii* infection.

Conversely, the seroprevalence of anti-*T. gondii* anti-IgG antibodies seroprevalence showed a slight increase from 37.81% to 38.54%. This increase was pronounced in rural areas (the highest was in the 31–35 age group), while in urban areas, we recorded a decrease.

In Dolj County, South-West Romania, the high prevalence of toxoplasmosis childbearing women suggests an urgent need to enhance screening and toxoplasmosis awareness. The notably higher prevalence of toxoplasmosis in smaller settlements around Craiova city compared to the city itself suggests that fertile women in rural areas are at higher risk.

This study also underscores the necessity for further investigation to augment our understanding of the factors that influence the prevalence of toxoplasmosis across various regions and populations. This could aid in the formulation of efficacious strategies for the prevention and control of this infection.

## Figures and Tables

**Table 1:** Demographic characteristics of the fertile age women tested for IgM anti-*T. gondii* antibodies.

	Group 1 *n* = 602	Group 2 *n* = 3014	*p*
Area of residence (*n*/%)			
Rural	206 (34.21%)	1704 (56.54)	<0.001 *
Urban	396 (65.79)	1310 (43.46%)	
Age (years)	27.79 (5.46)	27.62 (6.24)	0.537

*: Statistical significant result.

**Table 2:** Comparative seroprevalence of anti-*T. gondii* IgM antibodies between the two groups stratified by the area of residence and age group.

Variable	Group 1 2013–2016 (*n* = 602)	Group 2 2019–2022 (*n* = 3014)	*p* Value
TOTAL PREVALENCE	14/602 (2.32%)	32/3014 (1.06%)	0.012 *
AREA OF RESIDENCE			
Rural	6/206 (2.91%)	24/1704 (1.40%)	0.099
Urban	8/396 (2.02%)	8/1310 (0.61%)	0.011 *
AGE GROUP			
<20 years	3/57 (5.26%)	7/436 (1.60%)	0.065
21–25 years	3/145 (2.06%)	5/735 (0.68%)	0.109
26–30 years	4/239 (1.67%)	10/847 (1.18%)	0.553
31–35 years	2/105 (1.90%)	4/656 (0.60%)	0.160
>35 years	2/56 (3.57%)	6/340 (1.76%)	0.372
AREA OF RESIDENCE AND AGE GROUP			
Rural, <20 years	1/37 (2.70%)	6/333 (1.80%)	0.703
Rural, 21–25 years	1/61 (1.63%)	4/509 (0.78%)	0.500
Rural, 26–30 years	1/67 (1.49%)	7/433 (1.61%)	0.942
Rural, 31–35 years	2/29 (6.89%)	2/287 (0.69%)	0.004 *
Rural, >35 years	1/12 (8.33%)	5/142 (3.52%)	0.408
Urban, <20 years	2/20 (10.00%)	1/103 (0.97%)	0.017 *
Urban, 21–25 years	2/84 (2.38%)	1/226 (0.74%)	0.235
Urban, 26–30 years	3/172 (1.74%)	3/414 (0.72%)	0.263
Urban, 31–35 years	0/76 (0%)	2/369 (0.54%)	0.521
Urban, >35 years	1/44 (2.27%)	1/198 (0.50%)	0.234

*: Statistical significant result.

**Table 3:** Seroprevalence of anti-*T. gondii* IgM antibodies in the two groups according to demographic factors.

	Group 1 (2013–2016) (*n* = 602)									Group 2 (2019–2022) (*n* = 3014)								
	Area of Residence									Area of Residence								
	Rural			Urban			Total			Rural			Urban			Total		
	Neg.	Pos.	Total	Neg.	Pos.	Total	Neg.	Pos.	Total	Neg.	Pos.	Total	Neg.	Pos.	Total	Neg.	Pos.	Total
Age group																		
<20 years																		
No.	36	1	37	18	2	20	54	3	57	327	6	333	102	1	103	429	7	436
%	97.29	2.70	100	90.0	10.0	100	94.73	5.26	100	98.19	1.80	100	99.02	0.97	100	98.39	1.60	100
21–25 years																		
No.	60	1	61	82	2	84	142	3	145	505	4	509	225	1	226	730	5	735
%	98.36	1.63	100	97.61	2.38	100	97.93	2.06	100	99.21	0.78	100	99.55	0.44	100	99.31	0.68	100
26–30 years																		
No.	66	1	67	169	3	172	235	4	239	426	7	433	411	3	414	837	10	847
%	98.50	1.49	100	98.25	1.74	100	98.32	1.67	100	98.38	1.61	100	99.27	0.72	100	98.81	1.18	100
31–35 years																		
No.	27	2	29	76	0	76	103	2	105	285	2	287	367	2	369	652	4	656
%	93.10	6.89	100	100	0	100	98.09	1.90	100	99.30	0.69	100	99.45	0.54	100	99.39	0.60	100
>35 years																		
No.	11	1	12	43	1	44	54	2	56	137	5	142	197	1	198	334	6	340
%	91.66	8.33	100	97.72	2.27	100	96.42	3.57	100	96.47	3.52	100	99.49	0.50	100	98.23	1.76	100
Total																		
No.	200	6	206	388	8	396	588	14	602	1680	24	1704	1302	8	1310	2982	32	3014
%	97.08	2.91	100	97.97	2.02	100	97.67	2.32	100.00	98.59	1.40	100	99.38	0.61	100	98.94	1.06	100.00

**Table 4:** Demographic characteristics of the female subjects tested for IgG anti-*T. gondii* antibodies.

	Group 1 *n* = 603	Group 2 *n* = 2901	*p*
Area of residence (*n*/%)			
Rural	205 (34.00%)	1628 (56.12%)	<0.001 *
Urban	398 (66.00%)	1273 (43.88%)	
Age (years)	27.75 (5.42)	27.67 (6.23)	0.770

*: Statistical significant result.

**Table 5:** Comparative seroprevalence of anti-*T. gondii* IgG antibodies between the two groups, stratified by area of residence and age group.

Variable	Group 1 2013–2016 (*n* = 603)	Group 2 2019–2022 (*n* = 2901)	*p* Value
TOTAL PREVALENCE	228/603 (37.81%)	1118/2901 (38.54%)	0.737
AREA OF RESIDENCE			
RURAL	75/205 (36.59%)	691/1628 (42.44%)	0.109
URBAN	153/398 (38.44%)	427/1273 (33.54%)	0.073
AGE GROUP			
<20 YEARS	29/58 (50.00%)	132/416 (31.73%)	0.006 *
21–25 YEARS	56/142 (39.44%)	273/714 (38.24%)	0.788
26–30 YEARS	92/244 (37.70%)	313/824 (37.99%)	0.935
31–35 YEARS	32/105 (30.48%)	245/628 (39.01%)	0.095
>35 YEARS	19/54 (35.19%)	155/319 (48.59%)	0.068
AREA OF RESIDENCE AND AGE GROUP			
RURAL, <20 YEARS	19/37 (51.35)	99/315 (31.43)	0.015 *
RURAL, 21–25 YEARS	17/59 (28.81%)	189/494 (38.26%)	0.012 *
RURAL, 26–30 YEARS	26/68 (38.24%)	192/418 (45.93%)	0.237
RURAL, 31–35 YEARS	4/29 (13.79%)	129/269 (47.96%)	<0.001 *
RURAL, >35 YEARS	9/12 (75.00%)	82/132 (62.12%)	0.376
URBAN, <20 YEARS	10/21 (47.62%)	33/101 (32.67%)	0.192
URBAN, 21–25 YEARS	39/83 (46.99%)	84/220 (38.18%)	0.164
URBAN, 26–30 YEARS	66/176 (37.50%)	121/406 (29.80%)	0.068
URBAN, 31–35 YEARS	28/76 (36.84%)	116/359 (32.31%)	0.446
URBAN, >35 YEARS	10/42 (23.81%)	73/187 (39.04%)	0.063

*: Statistical significant result.

**Table 6:** Seroprevalence of anti-*T. gondii* IgG antibodies in the two groups according to demographic factors.

	Group 1 (2013–2016) (*n* = 603)									Group 2 (2019–2022) (*n* = 2901)								
	Area of Residence									Area of Residence								
	Rural			Urban			Total			Rural			Urban			Total		
	Neg.	Pos.	Total	Neg.	Pos.	Total	Neg.	Pos.	Total	Neg.	Pos.	Total	Neg.	Pos.	Total	Neg.	Pos.	Total
Age group																		
<20 years																		
No.	18	19	37	11	10	21	29	29	58	216	99	315	68	33	101	284	132	416
%	48.65	51.35	100.00	52.38	47.62	100.00	50.00	50.00	100.00	68.57	31.43	100.00	67.33	32.67	100.00	68.27	31.73	100.00
21–25 years																		
No.	42	17	59	44	39	83	86	56	142	305	189	494	136	84	220	441	273	714
%	71.19	28.81	100.00	53.01	46.99	100.00	60.56	39.44	100.00	61.74	38.26	100.00	61.82	38.18	100.00	61.76	38.24	100.00
26–30 years																		
No.	42	26	68	110	66	176	152	92	244	226	192	418	285	121	406	511	313	824
%	61.76	38.24	100.00	62.50	37.50	100.00	62.30	37.70	100.00	54.07	45.93	100.00	70.20	29.80	100.00	62.01	37.99	100.00
31–35 years																		
No.	25	4	29	48	28	76	73	32	105	140	129	269	243	116	359	383	245	628
%	86.21	13.79	100.00	63.16	36.84	100.00	69.52	30.48	100.00	52.04	47.96	100.00	67.69	32.31	100.00	60.99	39.01	100.00
>35 years																		
No.	3	9	12	32	10	42	35	19	54	50	82	132	114	73	187	164	155	319
%	25.00	75.00	100.00	76.19	23.81	100.00	64.81	35.19	100.00	37.88	62.12	100.00	60.96	39.04	100.00	51.41	48.59	100.00
Total																		
No.	130	75	205	245	153	398	375	228	603	937	691	1628	846	427	1273	1783	1118	2901
%	63.41	36.59	100.00	61.56	38.44	100.00	62.19	37.81	100.00	57.56	42.44	100.00	66.46	33.54	100.00	61.46	38.54	100.00

**Table 7:** Prevalence of IgM and IgG anti-*T. gondii* antibodies.

	Group 1 (2013–2016)			Group 2 (2019–2022)		
	Anti-*T. gondii* IgG (−)	Anti-*T. gondii* IgG (+)	Total	Anti-*T. gondii* IgG (−)	Anti-*T. gondii* IgG (+)	Total
ANTI-*T. GONDII* IGM (+)	12 (5.48%)	2 (0.53%)	14 (2.36%)	25 (2.25%)	1 (0.06%)	26 (0.90%)
ANTI-*T. GONDII* IGM (−)	207 (94.52%)	373 (99.47%)	580 (97.64%)	1086 (97.75%)	1771 (99.94%)	2857 (99.10%)
TOTAL	219 (100%)	375 (100%)	594 (100%)	1111 (100%)	1772 (100%)	2883 (100%)
ODDS	0.058	0.005	0.024	0.023	0.001	0.009
ODDS RATIO	10.81			40.77		
*P* VALUE	<0.001 *			<0.001 *		
95% CI	2.36–100.00			6.65–1674.49		

*: Statistical significant result.

**Table 8:** IgG Anti-*T. gondii* antibodies seroprevalence in childbearing age women—spread in Dolj county, Romania, 2019–2022.

Town	Seroprevalence of Anti-*T. gondii* IgG Antibodies	Town	Seroprevalence of Anti-*T. gondii* IgG Antibodies
Podari	58.97%	Coșoveni	37.14%
Sadova	57.58%	Bucovăț	36.36%
Segarcea	45.83%	Calafat	34.29%
Teasc	42.86%	Calopăr	33.33%
Filiași	41.94%	Malu Mare	32.00%
Desa	40.91%	Craiova	31.63%
Mârșani	40.91%	Dăbuleni	31.58%
Băilești	40.00%	Ciupercenii Noi	18.18%

## Data Availability

The data presented in this study are available on request from the corresponding author. The data are not publicly available due to patient personal data protection policy of the University and Hospital.
